# Multi-objective RGB-D fusion network for non-destructive strawberry trait assessment

**DOI:** 10.3389/fpls.2025.1564301

**Published:** 2025-03-12

**Authors:** Zhenzhen Cheng, Yifan Cheng, Bailing Miao, Tingting Fang, Shoufu Gong

**Affiliations:** ^1^ Department of Horticulture, Xinyang Agriculture and Forestry University, Xinyang, China; ^2^ Department of Optical and Electronic Information, Huazhong University of Science and Technology, Wuhan, China

**Keywords:** strawberry quality, fruit traits estimation, computer vision, deep learning, RGB-D modality fusion

## Abstract

Growing consumer demand for high-quality strawberries has highlighted the need for accurate, efficient, and non-destructive methods to assess key postharvest quality traits, such as weight, size uniformity, and quantity. This study proposes a multi-objective learning algorithm that leverages RGB-D multimodal information to estimate these quality metrics. The algorithm develops a fusion expert network architecture that maximizes the use of multimodal features while preserving the distinct details of each modality. Additionally, a novel Heritable Loss function is implemented to reduce redundancy and enhance model performance. Experimental results show that the coefficient of determination (R²) values ​​for weight, size uniformity and number are 0.94, 0.90 and 0.95 respectively. Ablation studies demonstrate the advantage of the architecture in multimodal, multi-task prediction accuracy. Compared to single-modality models, non-fusion branch networks, and attention-enhanced fusion models, our approach achieves enhanced performance across multi-task learning scenarios, providing more precise data for trait assessment and precision strawberry applications.

## 1 Introduction

Strawberries are highly valued for their delightful flavor, taste, and nutritional benefits (Hernández-Martínez et al., 2023; Liu et al., 2023; Gudowska et al., 2024). Over the past decade, global strawberry production has surged by 28%, exceeding 8.8 million tons (2011-2020, FAOSTAT). To remain competitive, producers must meet consumer demands for strawberries with uniform size and appealing weight—attributes closely associated with better flavor and nutritional value (Hopf et al., 2022; James et al., 2022; Miranda et al., 2023; Simkova et al., 2023). Accurate strawberry counting is also essential, especially in post-harvest evaluation, where precise assessments influence yield prediction and packaging strategies (Behera et al., 2020; Birania et al., 2022). However, current evaluation methods still rely on traditional techniques such as visual inspection, manual measurement, and weighing, which are not only destructive but also less accurate, labor-intensive, and time-consuming (Abebe et al., 2023; Jócsák et al., 2023; Azam et al., 2019). This underscores the pressing need for a rapid and non-destructive method to estimate strawberry weight, size uniformity, and quantity, enhancing both efficiency and accuracy.

The rise of computer vision technology has brought about revolutionary changes in the field of agricultural non-destructive testing (Mahanti et al., 2022; Sivaranjani et al., 2022; Ferrer-Ferrer et al., 2023). The adoption of numerous image processing algorithms has made it possible to rapidly and reliably assess crop traits. For instance, these methods have demonstrated significant effectiveness in evaluating citrus (Srivastava et al., 2020; Srivastava and Sadistap, 2022), mango (Pise and Upadhye, 2018; Mon and ZarAung, 2020), Grape (Underhill et al., 2020; Al-Saif et al., 2022; Zha et al., 2023), and apple (Sofu et al., 2016; Grabska et al., 2023) For strawberries, significant research has been conducted, particularly focused on evaluating external traits, such as fruit dimensions (Ishikawa et al., 2018; Nagamatsu et al., 2021; Zingaretti et al., 2021) and morphological traits (Feldmann et al., 2020), which are commonly used for grading or genetic pattern identification through machine learning algorithms (Oo and Aung, 2018; Rey-Serra et al., 2021). Basak et al. (2022) and Oliveira et al. (2023) employed linear regression, nonlinear regression, and support vector machine algorithms to construct a correlation model between image pixel count and strawberry weight, achieving a prediction accuracy of over 85%. Despite these promising results, the absence of 3D depth information in RGB images remains a concern. Because images capture only two dimensions while the real world exists in three-dimensional space, relying solely on 2D-pixel data can introduce inaccuracies in the size-weight relationship.

To address these limitations, expanding information dimensions has proven effective. With advances in image acquisition technologies, non-destructive testing (NDT) methods that integrate multimodal data (such as depth images, infrared images, and multispectral images) have become increasingly widespread (Xiang and Wang, 2023). Depth images, in particular, provide unique advantages by providing not only two-dimensional data but also depth information for each pixel. This additional spatial dimension is crucial for characterizing fruit physical properties, such as size (Lopes et al., 2022). Since the introduction of the low-cost Microsoft Kinect sensor in 2010, the prevalence of RGB-D sensors in computer vision has increased significantly (Fu et al., 2020).

With depth images as supplementary information, various multimodal information extraction and fusion architectures have been developed to optimize the use of RGB-D data. Building on this, the key challenge has become how to effectively utilize these multimodal data. Current RGB-D architectures generally fall into two categories. The first type, as demonstrated by Rong et al. (2023), employs a data-layer fusion strategy that treats multimodal data as indistinguishable multichannel inputs. This approach risks modal interference, potentially introducing noise or losing critical information when combining features, which can degrade model performance. The second approach, commonly referred to as a multi-branch architecture, utilizes at least two independent branches to separately learn features from each modality. For instance, Lin et al. (2022) developed a branched architecture regression network using RGB-D, allowing for the extraction of RGB, depth, and geometric features. Similarly, Zhang et al. (2022) developed a multi-stage branched self-correcting network for lettuce trait estimation, which uses RGB images for LW, DW, D, and LA estimation, and pseudocolor images derived from depth data for H estimation. While the multi-branch architecture mitigates the issue of modal interference by independently learning features from each modality, it introduces its challenges. A primary drawback is that, although it reduces the risk of information loss, it can lead to redundancy by duplicating similar features across branches. This redundancy may increase the model’s complexity and computational cost without proportionately improving performance. Furthermore, because the features are learned separately, this architecture often struggles to generate new insights or representations that are independent of the original modalities. In contrast to fusion-based methods, multi-branch architectures can struggle to fully exploit the complementary nature of multimodal data. A key reason why traditional methods fail to effectively handle modality interference is their inability to properly disentangle useful modality-specific information from cross-modal redundancy and noise. Early fusion methods, which directly concatenate RGB and depth features at the input level, often mix modality-specific characteristics, leading to feature entanglement and degraded discrimination power. keeping modalities separate until the decision stage inherently limits the model’s ability to capture rich inter-modal dependencies. As a result, these traditional approaches often face difficulties in fully leveraging the complementary nature of multimodal information while minimizing unwanted interference, which is crucial for effective RGB-D data fusion.

To overcome challenges such as modal interference and feature redundancy, this study designs a novel multimodal multi-task learning network. The core of the network leverages expert networks to independently extract features from each modality, preventing interference between modalities and allowing each expert network to focus on in-depth feature extraction specific to its modality. Additionally, to enhance the model’s representation capability, particularly in handling fused RGB and depth features, this paper introduces a new heritable loss function. This loss function adjusts the similarity between RGB features, depth features, and fused features, reducing linear correlations while improving model expressiveness and accuracy. Unlike traditional orthogonal loss, heritable loss retains intrinsic modality correlations, allowing the fused features to inherit both RGB and depth advantages while generating new, distinct representations. This approach effectively avoids the information loss caused by excessive decorrelation in traditional methods, improving adaptability in multimodal data fusion tasks. Extensive experiments on an RGB-D strawberry dataset demonstrate that this method surpasses existing approaches in accuracy, efficiency, and scalability.

## 2 Materials and methods

### 2.1 Dataset and preprocessing

#### 2.1.1 Sample collection of strawberries

**Figure 1:**
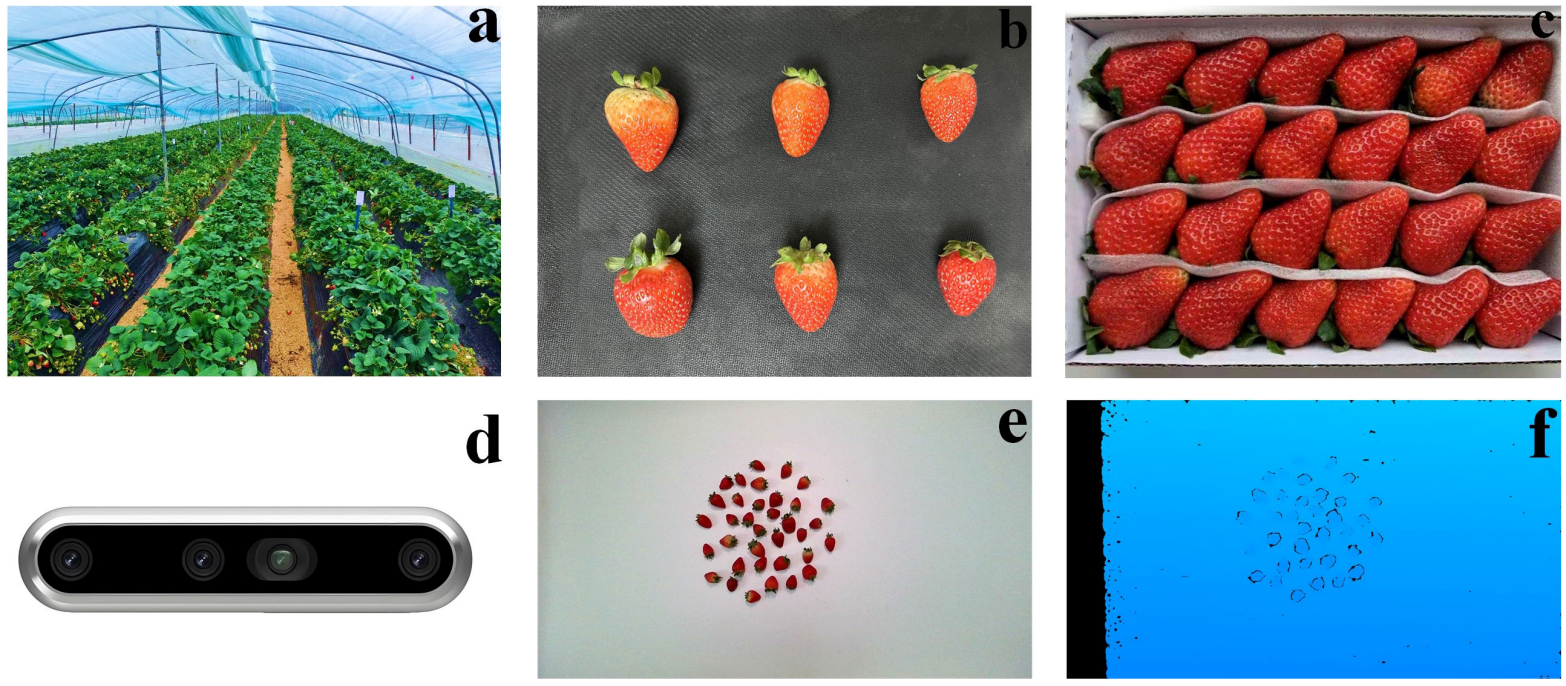
Strawberry cultivation, samples, and multimodal imaging. **(A)** strawberry demonstration garden greenhouse with rows of strawberry plants; **(B)** assorted strawberries of varying sizes collected in bulk; **(C)** uniform-sized premium strawberries neatly packaged in a box; **(D)** front view of the Intel RealSense D455 sensor; **(E)** RGB modal image of strawberries; **(F)** depth modal image of strawberries.

The strawberry samples were collected from the Strawberry Demonstration Garden located in Shisanliqiao Township, Xinyang City (**Figure 1A**). The garden employs a planting configuration where strawberry seedlings are spaced 15 cm apart, with a 35 cm pathway between rows, and extends 24 meters in length. The farm cultivates high-quality red strawberry varieties favored by local consumers. During the harvest season, from November to March, strawberries ripen in batches. Ripe strawberries awaiting packaging were specifically selected for the study. Each strawberry was carefully picked to maintain its integrity. The collected strawberries were then randomly divided into batches, each varying in number, shape, and size (**Figure 1B**). In addition to the randomly collected strawberries, a set of premium strawberries were purchased (**Figure 1C**). These strawberries, characterized by their uniform size and shape, were packaged into boxes, ensuring consistency in appearance and size, and representing the high-end market variety preferred by consumers. A total of 3740 strawberry samples were collected. The dataset was randomly divided into a training set (80%) and a test set (20%). To enhance model robustness, conventional data augmentation was applied to the training set, including random horizontal flipping and brightness adjustment.

The data collection process takes place at the Intelligent Equipment Laboratory of Xinyang Agriculture and Forestry College. The D455 sensor was used to photograph various batches of strawberries (**Figure 1D**). The Intel RealSense D455 is a depth camera introduced by Microsoft based on stereoscopic infrared sensing. Unlike traditional RGB cameras, the D455 captures detailed depth information, with a measurement range of 0.4 m to 6 m. The depth accuracy varies with distance, with an error of approximately ±2 mm at 1 m and ±6 mm at 4 m, making it suitable for long-distance applications in industrial settings. Using the D455 sensor, both RGB (**Figure 1E**) and depth (D) modal images (**Figure 1F**) were captured. The RGB modal image provides standard color-based visual data, while the depth modal image offers precise distance measurements from the sensor to each strawberry. In this study, the sensor was positioned at a height of 0.6 meters of 0°to accommodate the small size of the strawberry fruits. Since the D455 captures RGB images at a resolution of 1280×720 and depth images at 640×480, bilinear interpolation was applied to upsample depth images to match the resolution of RGB images before feature extraction. Additionally, Gaussian filtering was employed to reduce potential noise introduced by interpolation, further improving the quality of depth data integration. Detailed specifications and additional information about the D455 can be found on the manufacturer’s website: https://www.intelrealsense.com/depth-camera-d455/.

#### 2.1.2 Measurement of strawberry traits

Simultaneously with image capture, the number of strawberries in each batch, their respective fresh weights, and their size uniformity were recorded. Each batch was collected by trained personnel under consistent environmental conditions, including controlled light, temperature, and humidity. The number of strawberries was manually counted, and their fresh weights were measured using a digital balance (model: LQC-50001, manufacturer: Jiangsu LENQI Company, China). Before each measurement, the balance was calibrated to zero, and then each strawberry was individually placed on the weighing plate. The fresh weight of each strawberry was recorded in grams, following the operating instructions and precautions outlined in the balance’s manual. Each strawberry was weighed multiple times, and the average was used as the final fresh weight.

Size uniformity was assessed by calculating the coefficient of variation of the strawberry aspect ratio. The aspect ratio, which describes the shape of a strawberry, reflects its oval form. By calculating the coefficient of variation of the strawberries’ length-to-width ratios, we quantified the degree of size variation within each batch. The coefficient of variation is the standard deviation divided by the mean, expressed as a percentage. This metric accounts for both the range of size variation and the absolute size values, providing a more comprehensive assessment of size uniformity than other metrics like standard deviation or percentage error. The coefficient of variation (CV) is calculated using Equation 1:


(1) CV=∑i=1n(xi−x¯2)nx¯×100%

Where *n* is the number of strawberries in the batch; *x_i_* represents the size of the *i* th strawberry; $\bar{x}$ represents the mean size of the strawberries in the batch.

### 2.2 Proposed method

**Figure 2:**
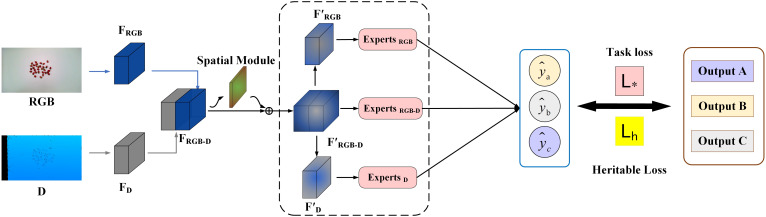
Overview of the proposed network.

This paper presents a model, called the Fusion Expert Network, designed to estimate the strawberries’ traits. As shown in **Figure 2**, the network first extracts shallow features from RGB and depth images using deep separable convolutional layers. Once feature extraction is complete, these features are combined and an attention fusion module generates masks to highlight areas of interest in the image, such as regions containing strawberries. The features are then separated into masked RGB features, masked RGB-D fusion features, and masked depth features. These three feature sets are assigned to independent branches, each processed by a dedicated expert network, reducing interference between modalities. In the final stage, a “heritable loss” function is introduced to adjust the similarity between RGB, depth, and fused features to reduce linear correlations and improve the model’s expressiveness and accuracy.

#### 2.2.1 Single-modal feature extraction module

The single-modal feature extraction module is the first stage of our proposed framework, designed to independently extract informative features from RGB and depth images. This module utilizes two identical depthwise separable convolutional layers to process the RGB and depth images separately, ensuring that there are no shared parameters between the modalities. This approach allows for the extraction of low-level features that capture essential visual cues specific to each modality. Depthwise separable convolutions are employed to simplify computations at this stage, as the focus is on extracting shallow features rather than deeper ones. A 3x3 kernel is used to emphasize local feature extraction, while a stride of 1 preserves the spatial resolution of the input images, ensuring the fidelity of the extracted features.

**Figure 3:**
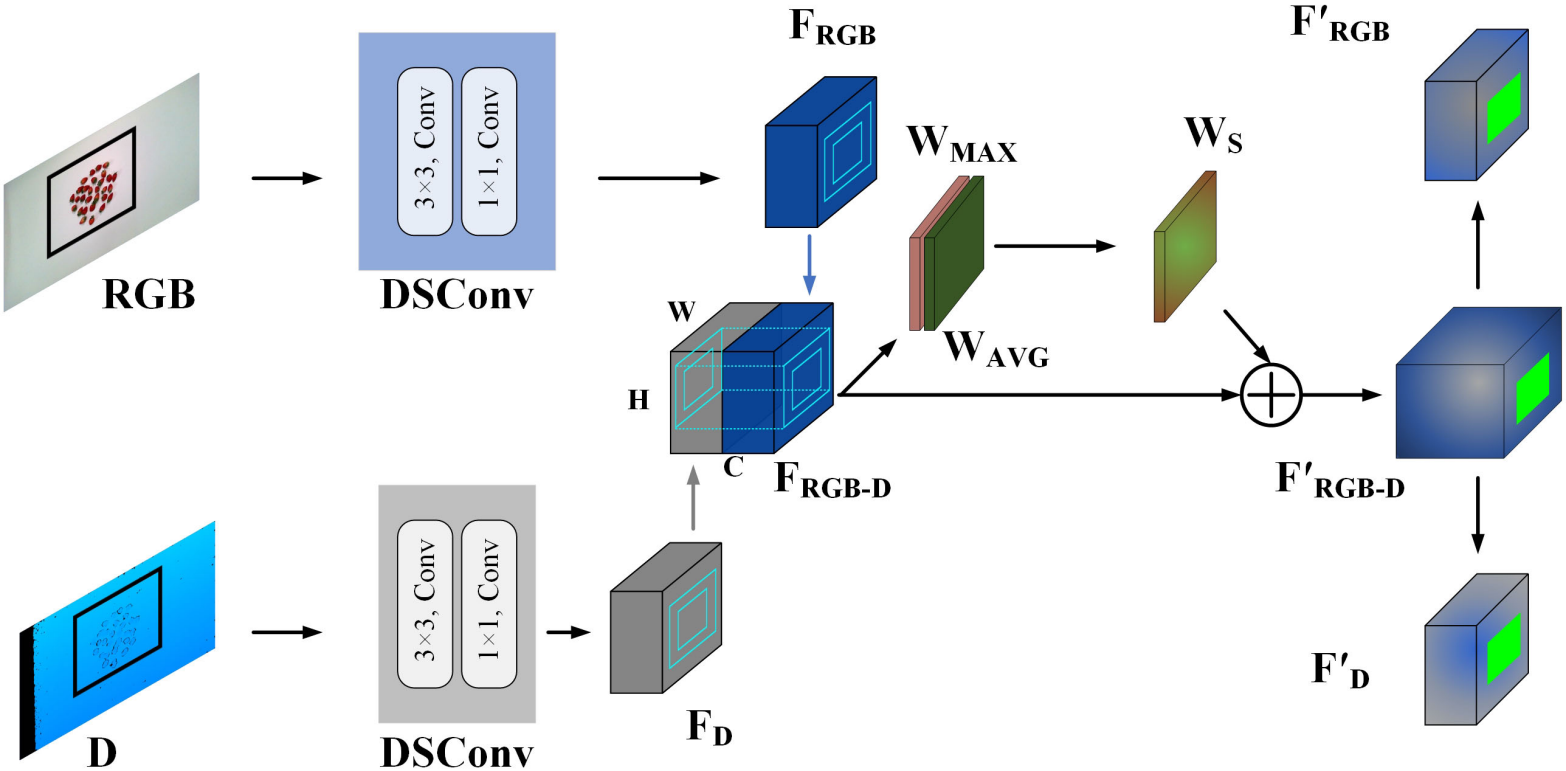
The structure of the single-modal feature extraction module.


After extracting shallow features, the RGB and depth image features are concatenated to form the combined feature F_RGB-D_, as shown in **Figure 3**. It can be represented as Equation 2:

(2) $${\text{F}}_{\text{RGB}-\text{D}}=\left[{\text{F}}_{\text{RGB}},{\text{F}}_{\text{D}}\right]$$

The resulting feature F_RGB-D_ is then input into the attention fusion module, where a spatial attention mechanism is employed to enhance the model’s focus on target areas. Specifically, F_RGB-D_ undergoes max pooling (*W _MAX_*) and average pooling (*W _AVG_*) along the channel dimension. These pooled feature maps are concatenated, followed by a dimensionality reduction step using a 7×7 convolutional layer to reduce the number of channels to one. The Sigmoid activation function is then applied to generate spatial weight coefficients, calculated as Equation 3:


(3) $$\begin{array}{c} {\text{W}}_{\text{S}}=\sigma {\text{f}}^{7\times 7}\left[\text{AvgPool}\left({\text{F}}_{\text{RGB}-\text{D}}\right);\text{MaxPool}\left({\text{F}}_{\text{RGB}-\text{D}}\right)\right]\\ =\sigma {\text{f}}^{7\times 7}\left[{\text{W}}_{\text{AVG}};{\text{W}}_{\text{MAX}}\right] \end{array}$$

where σ denotes the sigmoid function and f 7×7 represents the convolution layer with a kernel size of 7 × 7.

These coefficients are then used in an element-wise multiplication with the input feature map, resulting in the final attention-weighted feature, denoted as F’_RGB-D_. F’_RGB-D_ is subsequently split along the channels to extract branch-specific features corresponding to different modalities. The split features are classified into three categories: masked RGB features, masked RGB-D fusion features, and masked Depth features. This separation allows the attention mechanism to guide the model’s focus toward relevant regions within each modality, enabling the subsequent specialized expert networks to process these features more efficiently.

#### 2.2.2 Three expert networks

Since different strawberry traits (weight, size uniformity, and number) may rely on distinct image features, we have designed a network comprising three experts: Expert_RGB_, Expert_RGB-D_, and Expert_D_. Each expert is dedicated to processing a specific modality of information. The expert networks are based on the ResNet18 architecture, a widely used and powerful convolutional neural network known for its effectiveness in image processing tasks (He et al., 2015). Compared to deeper architectures, ResNet-18 provides sufficient representational power while maintaining lower computational cost, making it well-suited for our multimodal framework. Additionally, its residual connections help mitigate gradient vanishing issues, ensuring stable training even with limited dataset size. These characteristics make ResNet-18 an effective choice for extracting modality-specific features in our expert network design. The detailed structure is outlined in **Table 1**.

**Table 1:** The single-expert networks architecture.

Layer	Kernel Size	Stride	Padding	Output Size	Output Channels
Conv1	7x7	2	3	112x112	64
Max Pooling	3x3	2	1	56x56	64
Conv2	3x3	1	1	56x56	64
Conv3	3x3	2	1	28x28	128
Conv4	3x3	2	1	14x14	256
Conv5	3x3	2	1	7x7	512

**Figure 4:**
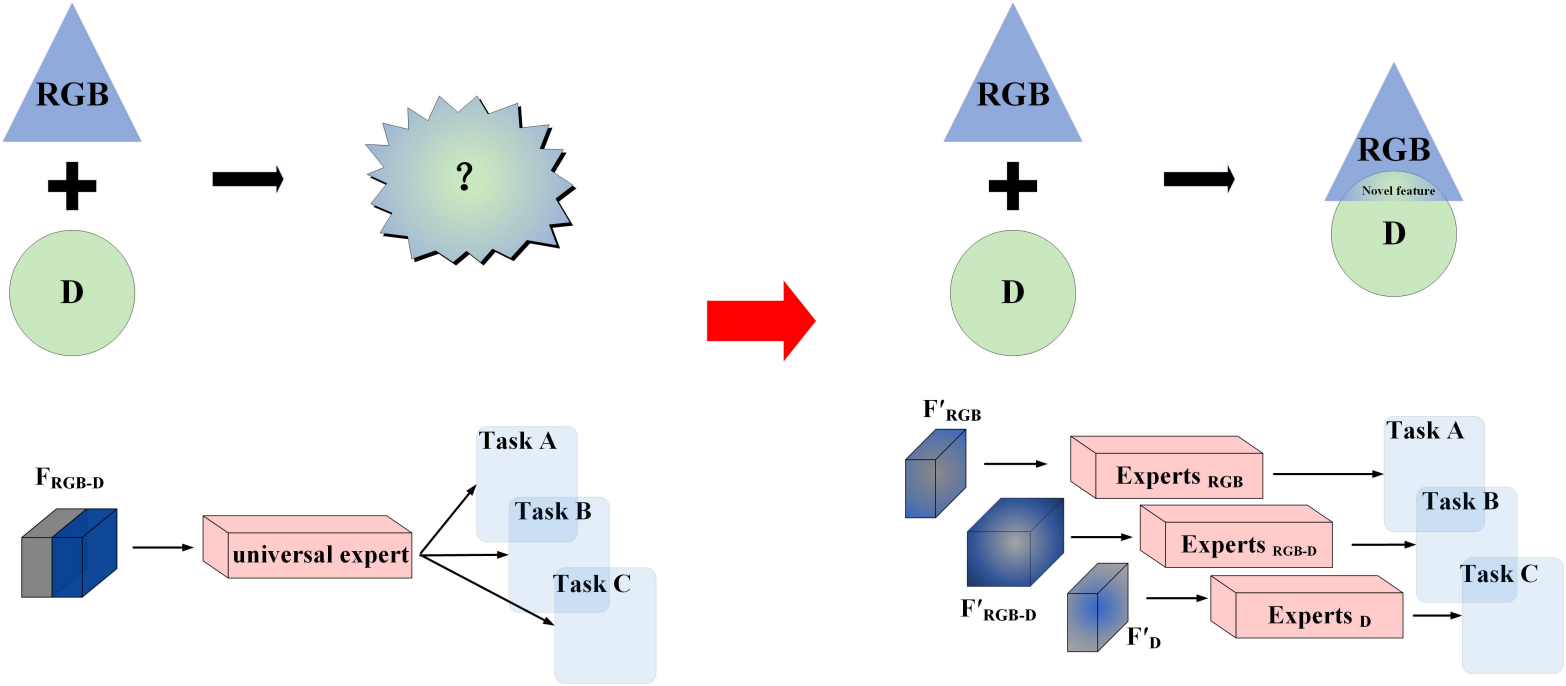
Illustration of multimodal information processing.

As illustrated in **Figure 4**, two different methods of feature fusion are compared to emphasize the necessity of our approach. The first method, shown on the left, represents a simple fusion of features from RGB and depth images processed through a ‘general network’. This basic method struggles to capture complex interactions between modalities, limiting its ability to generate meaningful new information. In contrast, the expert network approach on the right assigns features from different modalities to specialized networks. By processing these features independently, our method reduces information interference between modalities and better preserves the original data. This allows the expert networks to use complementary information more effectively, producing novel features.

#### 2.2.3 Heritable loss

**Figure 5:**
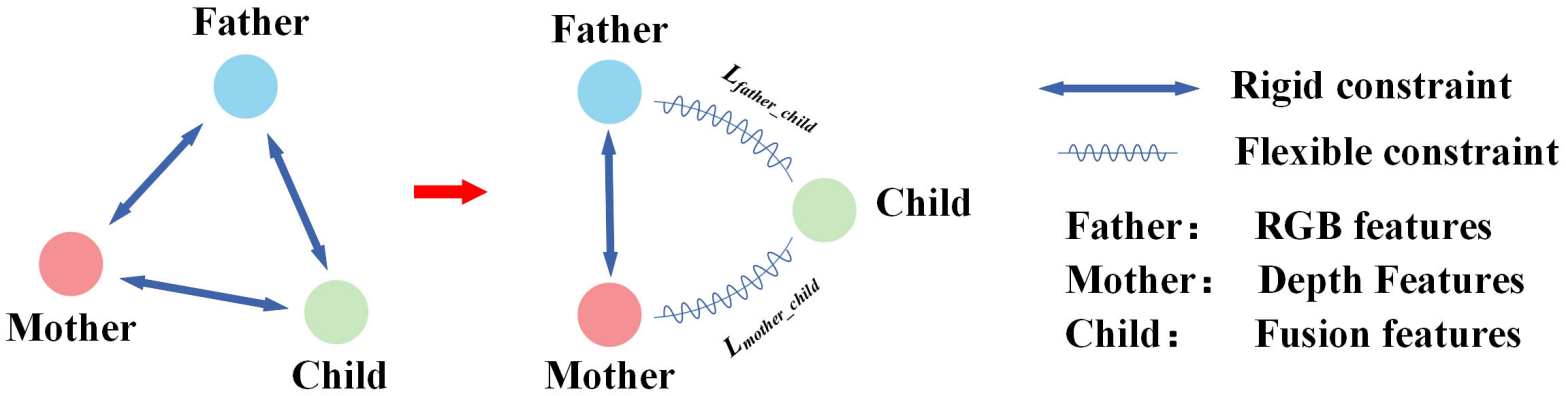
Learning with heritable loss.

To fully leverage the advantages of multimodal data fusion, it is crucial to extract new and unique fused features that differ from the original RGB and depth image features. Traditional multi-source fusion models often use orthogonal loss (Ranasinghe et al., 2021) to minimize correlations between feature sets, thereby enforcing feature independence (as shown in **Figure 5**). While this approach helps generate novel fused information and reduces linear correlations to avoid redundancy, it imposes a strong “hard constraint” on the newly generated features. This excessive restriction can hinder the final model’s convergence, as the newly generated features and the original modality features may not always adhere to strict orthogonality.

To address this issue, we propose a new loss function, termed “heritable loss.” This function introduces an adjustable threshold (τ) rather than simply minimizing the loss. This design is motivated by three key factors: (1) preventing overfitting by avoiding unnecessary optimization of low-impact features, thereby improving the model’s generalization; (2) enhancing feature selection by ensuring that only the most informative fused features contribute to the final prediction; and (3) improving computational efficiency by reducing redundant gradient updates. These advantages collectively ensure that the heritable loss flexible control over the similarity between RGB features, depth features, and fused features. This “flexible constraint” not only applies orthogonal constraints but also provides enough freedom for feature generation, thereby avoiding the negative effects of overly strict constraints.

The term “Heritable Loss” is used to convey the idea that certain characteristics of the fused features (the “child”) are inherently inherited from both the RGB (the “father”) and Depth (the “mother”) features. The child features operate with some degree of freedom within the constrained space created by the orthogonalization of the parent features. Within this space, the child features naturally find an optimal balance, compensating for any excessive orthogonalization while maintaining their uniqueness. **Figure 5** illustrates the comparison between the heritable loss and the traditional orthogonal loss. The left panel depicts the traditional orthogonalization mechanism, where the relationship between the “father,” “mother,” and “child” features is constrained into a triangular arrangement, enforcing independence and orthogonality among the features. In contrast, the right panel shows the relationship under our proposed heritable loss. In this case, the “child” feature is positioned on two arcs, oriented towards the “father” (RGB) and “mother” (Depth) features. The relationship between them is controlled by the following mechanisms:

The heritable loss function is composed of two primary components: L*_parent_* to measure the similarity between the RGB and Depth features, and L*_child_* to regulate the disparity between the fused features (RGB + Depth) and both the RGB and Depth features. It can be formulated as Equation 4:


(4) $${\text{L}}_{\text{heritable}}={\text{L}}_{\text{parent}}+{\text{L}}_{\text{chilad}}$$

Where:

*L_parent_*: This is a cosine similarity-based loss that quantifies the similarity between the RGB features (father) and Depth features (mother). It can be formulated as Equation 5:


(5) $${\text{L}}_{\text{parent}}=\text{Cos}\_\text{sim}\left(\text{father},\text{mother}\right)$$

*L_child_*: This component introduces a threshold τ to control the disparity between the fused features and the RGB and Depth features. It can be formulated as Equations 6–8:


(6) $${\text{L}}_{\text{child}}=\text{ReLU}\left(\left|{\text{L}}_{\text{fathe}{\text{r}}_{\text{child}}}-{\text{L}}_{\text{mothe}{\text{r}}_{\text{child}}}\right|-\text{τ}\right)$$

(7) $${\text{L}}_{\text{father}\_\text{child}}=\text{Cos}\_\text{sim}\left(\text{father},\text{child}\right)$$

(8) $${\text{L}}_{\text{mother}\_\text{child}}=\text{Cos}\_\text{sim}\left(\text{mother},\text{child}\right)$$

Here, ReLU is applied to ensure that the loss remains non-negative, preventing negative penalties during training. *L_father-child_* represents the cosine similarity between the fused features (child) and the RGB features (father), and *L_mother-child_* represents the cosine similarity between the fused features and the Depth features (mother). τ is a hyperparameter that controls the threshold for similarity differences, allowing for flexibility in determining how much similarity between features is acceptable.

When ∣*L_father-child_*−*L_mother-child_* ∣>τ, *L_child_* equals this difference minus the threshold, resulting in a positive loss value. This positive loss encourages the model to reduce the difference in similarity between the fused features and the RGB and Depth features.

Conversely, when ∣*L_father-child_*−*L_mother-child_* ∣≤τ, the value of *L_child_* becomes zero, meaning the model is not penalized and the constraint does not affect the training process in this case.

By incorporating the concept of the heritable loss term, the total loss function can be designed to effectively guide the training process. The heritable loss (L_heritable_)is combined with the task-specific loss (L_task_) to create the total loss function (L_total_). This is achieved by introducing a weight λ to balance the influence of each component. The total loss function is computed as Equations 9, 10:


(9) $${\text{L}}_{\text{total}}={\text{L}}_{\text{task}}+\text{λ}{\text{L}}_{\text{heritable}}$$

(10) $${\text{L}}_{\text{task}}=\frac{1}{\text{N}}\sum_{\text{i}=1}^{\text{N}}({\left({\text{y}}_{\text{A}i}-{\hat{\text{y}}}_{\text{A}i}\right)}^{2}+\left({\text{y}}_{\text{B}i}-{\hat{\text{y}}}_{\text{B}i}\right)+\left({\text{y}}_{\text{C}i}-{\hat{\text{y}}}_{\text{C}i}{)}^{2}\right)$$

Where λ is a weight hyperparameter used to adjust the importance between heritable loss and task loss. *N* is the total number of samples in your dataset; *y_A,_ y_B,_ y_C_* are the ground truth values for sample s for sample *i* for TaskA, TaskB, and TaskC, respectively;. ${\hat{y}}_{A}$ ., ${\hat{y}}_{B}$ , ${\hat{y}}_{C}$ are the predicted values for sample *i* for TaskA, TaskB, and TaskC, respectively.

## 3 Experiments and results

### 3.1 Experimental setup

The experiment was conducted on a Linux workstation running Ubuntu 16.04 LTS/Linux system, equipped with an NVIDIA RTX3060Ti running with CUDA 12.4 acceleration. The software environment included Python 3.7 and PyTorch 1.8.0, coupled with CUDNN 8.0. The Adam optimizer was used with a 0.001 learning rate, 0.0001 weight decay, 0.9 momentum, batch size of 2, and 200 epochs.

We evaluated the model’s performance across multiple dimensions, focusing particularly on its predictive accuracy in estimating strawberry weight, size uniformity, and number. Additionally, ablation experiments were conducted to isolate and assess the contribution of each component to the model’s overall effectiveness. To quantify these evaluations, several regression metrics were employed, including the Normalized Root Mean Square Error (NRMSE), Normalized Root Mean Square Error of Prediction (NRMSEP), and the coefficient of determination (R²). These metrics were calculated using Equations 11–15:


(11) RMSE=∑i=1n(yi(train)−y^i(train))2n

(12) $$\text{NRMSE}=\frac{\text{RMSE}}{{\bar{\text{y}}}^{\left(\text{train}\right)}}$$

(13) RMSE=∑i=1m(yi(test)−y^i(test))2m

(14) $$\text{NRMSE}=\frac{\text{RMSE}}{{\bar{\text{y}}}^{\left(\text{test}\right)}}$$

(15) $${\text{R}}^{2}=1-\frac{\sum_{\text{i}=1}^{\text{m}}{\left({\text{y}}_{i}^{\left(\text{test}\right)}-{\hat{\text{y}}}_{\text{i}}^{\left(\text{test}\right)}\right)}^{2}}{\sum_{\text{i}=1}^{\text{m}}{\left({\text{y}}_{i}^{\left(\text{test}\right)}-{\bar{\text{y}}}_{\text{i}}^{\left(\text{test}\right)}\right)}^{2}}$$

where *n* is the number of samples in the training set, *y_i_^(train)^* and ${\hat{y}}_{i}^{\left(train\right)}$ denote the actual and predicted values for each sample from the training set. *m* is the number of samples in the test set, *y_i_^(test)^* and ${\hat{y}}_{i}^{\left(test\right)}$ ) denote actual and predicted values from the test set. ${\bar{y}}_{i}^{\left(train\right)}$ and. ${\bar{y}}_{i}^{\left(test\right)}$ is the mean of actual values in the training test and test set respectively.

### 3.2 Estimation of strawberry traits

**Figure 6:**
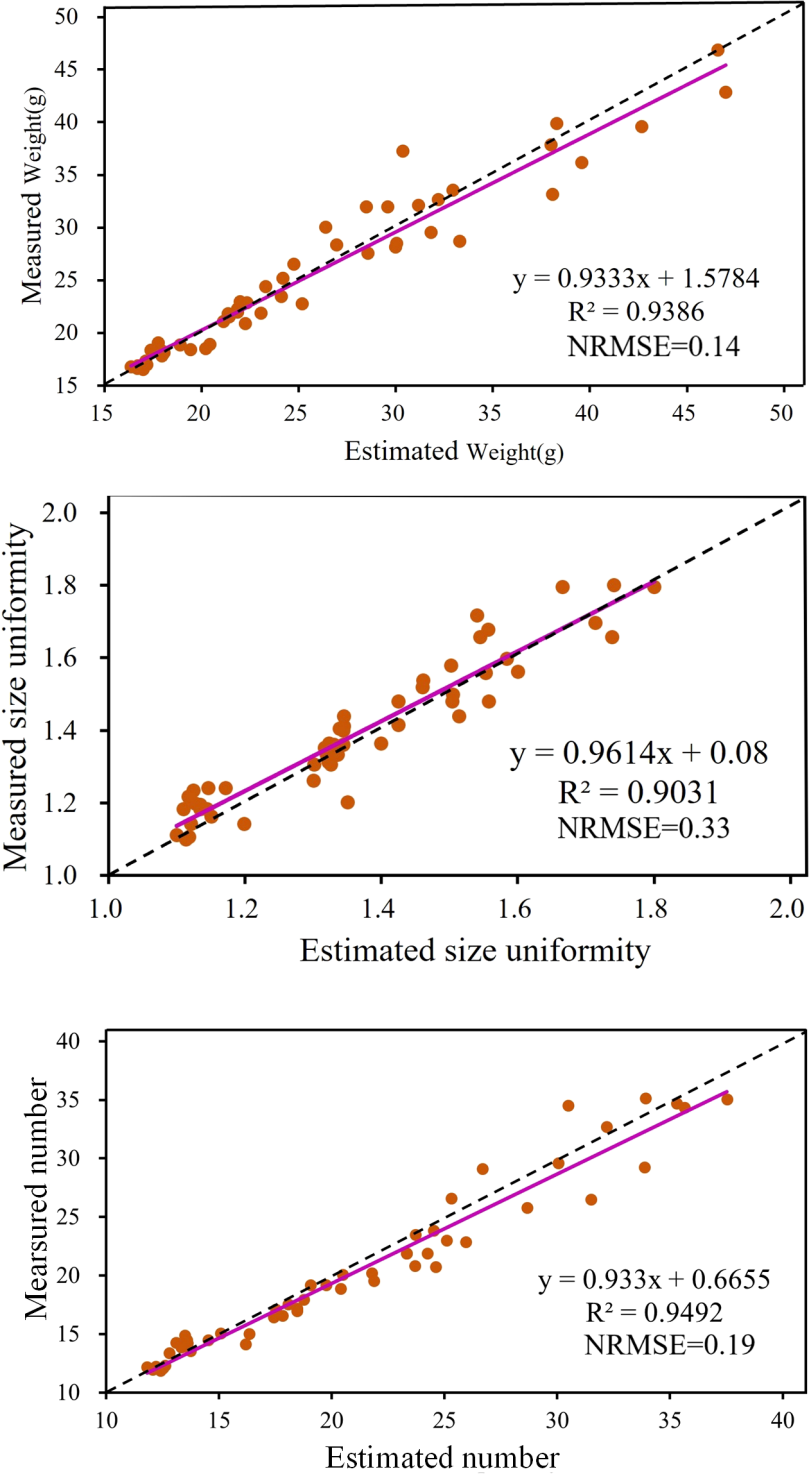
Estimation results of strawberry traits: weight, size uniformity, and number.

To evaluate the performance of the proposed model, a validation set of 50 images was used to test the trained model. **Figure 6** illustrates the relationship between measured and estimated values, where the horizontal axis represents the measured values and the vertical axis represents the model estimates. The magenta curve represents the relationship derived from least squares fitting. The lower right corner of each plot shows the evaluation metrics (R² and NRMSE) and the equation for the fitted curve. The R² value (ranging from 0 to 1) measures model quality, with higher values indicating a stronger linear correlation between estimated and measured values, reflecting better model performance. Conversely, a lower NRMSE value indicates smaller prediction errors relative to the data range, indicating higher prediction accuracy. The closeness of the fitted curve to the 45-degree dashed line provides an intuitive measure of how well the predictions match the actual measurements. The closer the curve, the better the agreement between predictions and actuals.

As shown in **Figure 6**, the R² values for weight, size uniformity, and number of strawberries are 0.94, 0.90, and 0.95, respectively. These high R² values demonstrate the model’s robust predictive capabilities across all three traits. Correspondingly, the NRMSE values are 0.14 for weight, 0.33 for size uniformity, and 0.19 for number. The lower NRMSE values for weight and number, compared to size uniformity, suggest that the model provides more precise predictions for these traits. In summary, the results quantitatively demonstrate that the proposed model achieves high prediction accuracy, particularly in estimating weight and number, with lower relative prediction errors.

### 3.3 Ablation experiment

The ablation strategy study of this experiment includes two parts: single component ablation and combination ablation experiments. The single component ablation focuses on removing key components of the model individually, such as the attention mechanism, expert network, and loss function, to evaluate the independent contribution of each component to the model performance. The combination ablation further examines the complementarity and overall impact on the model when two or more components are removed together. In particular, since the heritable loss relies on the presence of multiple experts, the evaluation of its effectiveness in the ablation experiments must account for the integrity of the expert network. The detailed experimental design and the obtained results are shown in **Table 2**, where A represents the attention mechanism, E represents the expert network, and L_h_ represents the heritable loss function.

**Table 2:** Model ablation experiment.

A	E	L_h_	Weight/g			Size uniformity			Number		
			NRMSE	NRMSEP	R^2^	NRMSE	NRMSEP	R^2^	NRMSE	NRMSEP	R^2^
			0.24	0.3	0.84	0.42	0.46	0.78	0.28	0.3	0.86
**√**			0.24	0.29	0.85	0.4	0.45	0.8	0.24	0.27	0.91
	**√**		0.2	0.22	0.89	0.38	0.42	0.83	0.26	0.32	0.88
**√**	**√**		0.18	0.22	0.88	0.37	0.4	0.85	0.21	0.24	0.92
	**√**	**√**	0.16	0.2	0.91	0.34	0.37	0.88	0.24	0.29	0.88
**√**	**√**	**√**	0.14	0.15	0.94	0.33	0.36	0.9	0.19	0.21	0.95

The A mechanism (attention mechanism) performs particularly well on counting tasks. Compared with the model with the E mechanism (expert network) added alone, the NRMSE, NRMSEP, and R² indicators of the model added with the A mechanism on the counting task have been significantly improved. Additionally, the performance of the model with the A mechanism and the model with both A and E mechanisms has been significantly improved compared with the baseline network. Furthermore, the model with the A and E mechanisms performs better than the model with the E and L_h_ mechanisms. The E mechanism has a significant improvement on all tasks. Compared with the baseline network, after adding the E mechanism, the NRMSE and NRMSEP of each task decreased, and the R² increased. For example, in the size uniformity task, the NRMSE decreased from 0.42 to 0.38, the NRMSEP decreased from 0.46 to 0.42, and the R² increased from 0.78 to 0.83. The L_h_ mechanism (heritable loss function) further optimizes the effect of the E mechanism and improves the feature extraction ability and redundancy removal of the model. Especially in the weight task, the NRMSE and NRMSEP are significantly reduced, and the R² is increased to 0.94.

The A+E+L_h_ combination demonstrates the best performance across all tasks. In weight estimation, this combination reduces NRMSE to 0.14, NRMSEP to 0.15, and increases R² to 0.94 outperforming other models significantly. These findings indicate that the combination of the A mechanism, E mechanism, and L_h_ mechanism more effectively extracts and fuses multimodal key features, thereby improving prediction accuracy. Although the improvement in size uniformity evaluation is modest, the precise feature separation achieved by this combination enhances model performance in more complex tasks. In the number detection task, the A+E+ L_h_ combination achieves an NRMSE of 0.19, an NRMSEP of 0.21, and an R² of 0.95. This demonstrates the synergistic effect of the A mechanism and L_h_ mechanism in guiding the E mechanism to manage multi-object scenes, enhancing both the robustness and accuracy of the predictions.

In conclusion, the A+E+ L_h_ combination highlights the high potential of the model in multi-task learning. Among the components, the expert network plays a critical role in enhancing performance across all tasks, leveraging deep learning to extract multi-level features. The attention mechanism primarily improves the model’s focus on local features, especially in weight and number estimation tasks. Finally, heritable loss effectively reduces feature redundancy and enhances the model’s generalization capacity.

### 3.4 Expert contribution and feature learning analysis

**Figure 7:**
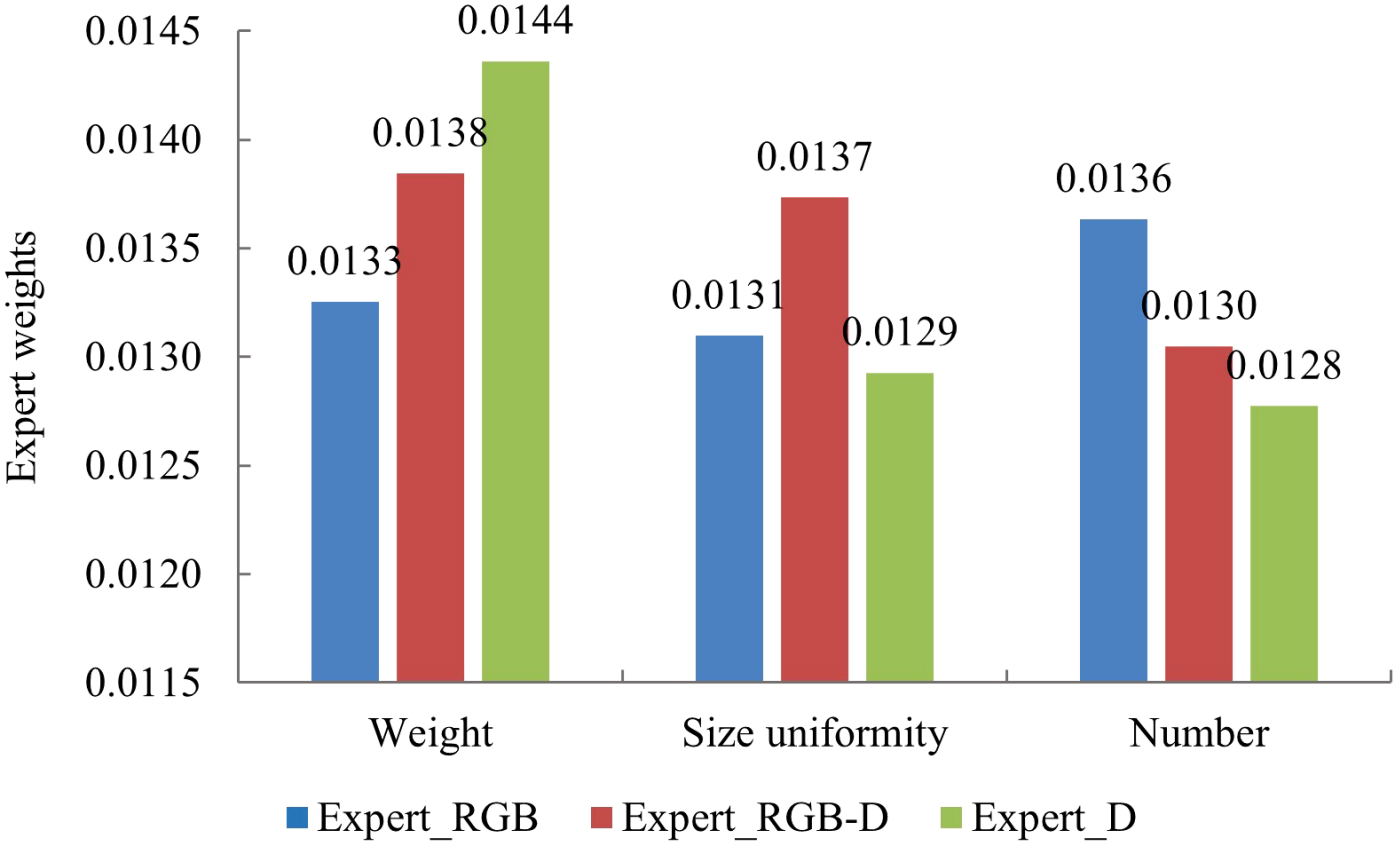
Histogram of expert contribution weights.

To further investigate and validate the effectiveness of expert networks in assessing the traits of strawberries, this paper compares and analyzes the weight contributions of three expert networks across different tasks, as illustrated in **Figure 7**. The results reveal distinct differences in the contributions of each expert network to various outputs. Specifically, in strawberry weight estimation, the Expert_D_ network shows the greatest contribution, with a weight of 0.0144. In contrast, the Expert_RGB-D_ network demonstrates the highest contribution to size uniformity estimation, with a weight of 0.0137. For number estimation, the Expert_RGB_ network contributes most significantly, with a weight of 0.0136. The differences in their contributions suggest that the distinct modal features captured by each Expert have varying levels of expressiveness. For instance, in the weight estimation task, the superior performance of the Expert_D_ network can be attributed to the fact that weight is closely related to the volume and shape of the strawberry, while depth image modality can effectively capture these geometric and 3D structural properties. In contrast, in number estimation, the higher contribution of the Expert_RGB_ network may stem from its exploitation of color and texture features, which are most prominent in RGB images and are crucial for counting and distinguishing individual strawberries. For size uniformity estimation, which requires an integrated assessment of both color and geometric features, the Expert_RGB-D_ network provides an effective fusion of RGB and depth information.

**Figure 8:**
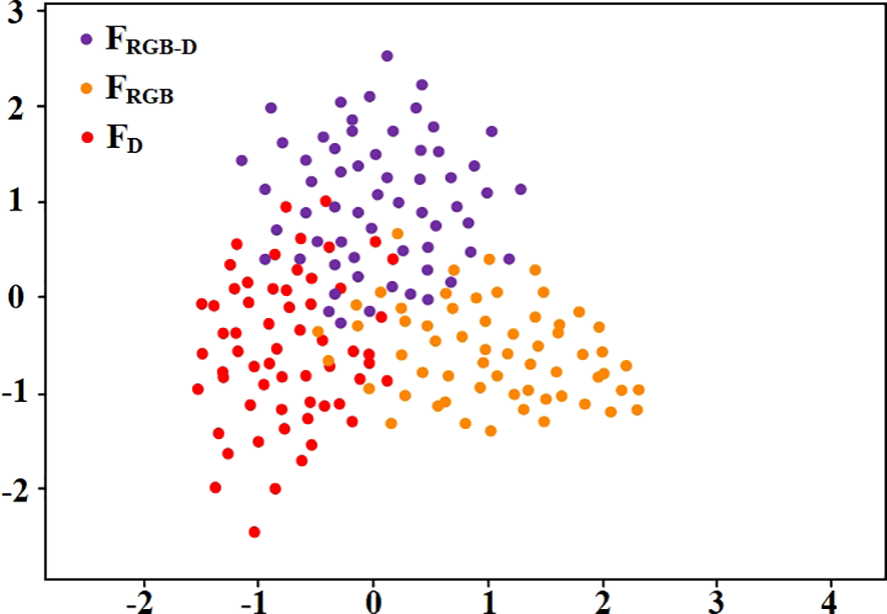
Clustered scatter plot of heritable loss impact on features.

To assess the effectiveness of the heritable loss term, we applied principal component analysis (PCA) to reduce feature dimensionality, subsequently visualizing the outcomes in a PCA feature clustering diagram, as depicted in **Figure 8**. In this diagram, the features derived from RGB images, RGB-D images, and depth images are represented by purple, orange, and red dots, respectively, and are denoted as F_RGB-D_, F_RGB_, and F_D_. The figure clearly illustrates that features corresponding to the same modality exhibit a pronounced clustering in the feature space. This clustering indicates high internal consistency and stability in the model’s feature extraction process, demonstrating the effectiveness of the feature extraction network. Notably, the clear separation of features from different modalities in the feature space indicates that the heritable loss has significantly reduced their linear correlations. This reduction enhances feature orthogonality and independence in high-dimensional space, effectively minimizing information overlap and redundancy, and improving the model’s ability to leverage multimodal information.

### 3.5 Comparative experiment

Given that our proposed method targets multimodal data fusion and multi-task learning scenarios, we conducted a comprehensive comparison with several alternative models. These comparison models were task-adapted to predict strawberry weight, size uniformity, and number. The models included in this comparison are: Single-Modal Models (He et al., 2015): This method utilizes only one type of input, either RGB images or depth images, without integrating data from multiple modalities. Branch Network Models Without Feature Fusion (Lin et al., 2022): This approach employs a multi-branch structure to handle different modalities independently. However, instead of sophisticated feature fusion, it simply concatenates the outputs of each branch. Attention-enhanced fusion model (Sun et al., 2022): Instead of processing the native RGB and depth (D) information directly, this method applies an attention mechanism to the RGB data before fusing it with the depth information. Mixture of Experts (MoE) Model (Ma et al., 2018): this model utilizes multiple expert networks, each specialized for a specific task or data pattern. The expert weights are dynamically adjusted by a gating network.

As shown in **Table 3**, The RGB model performs poorly in predicting size uniformity prediction due to its inability to capture the three-dimensional structural information of strawberries. Although the depth model performs well in other areas, it performs relatively poorly in number prediction because it lacks the necessary texture information. While the branch network can handle each modality independently, it doesn’t do as well in multimodal collaborative tasks because of the lack of interaction between features, resulting in less accurate predictions compared to the fusion model. The fusion model, which integrates features through an attention mechanism, outperforms the branch network in task prediction, particularly in tasks where multimodal collaboration is crucial, such as size uniformity and weight. The Mixture of Experts (MoE) model excels at handling the interaction and fusion of multimodal information. However, due to the dynamic selection instability of the gating mechanism, its performance in simple regression tasks slightly degrades, resulting in slightly lower accuracy in size uniformity and number prediction compared to the multimodal fusion model. In the early design stages of the proposed model, we observed that the gating mechanism is better suited for complex scenarios involving both regression and classification tasks than for single regression tasks. In contrast, our proposed method outperforms other models in all tasks, achieving higher R² values in strawberry weight, size uniformity, and number prediction.

**Table 3:** Comparison results in terms of R².

Model Type		R^2^		
		Weight/g	Size uniformity	Number
Single-modal	RGB	0.75	0.72	0.83
	depth	0.80	0.74	0.76
non-fusion branch network		0.80	0.76	0.85
Attention-enhanced fusion model		0.84	0.82	0.89
Mixture of Experts (MoE) Model		0.86	0.80	0.88
Proposed method		0.94	0.90	0.95

## 4 Conclusion

The goal of this study was to efficiently and accurately estimate weight, size uniformity, and number of strawberries. We proposed a multimodal multitask learning fusion expert network model. This method was able to utilize information from different modalities while preserving the unique information of each original modality through a multi-branch and single-path fusion strategy. Specifically, the model first extracts shallow features from RGB and depth images using depthwise separable convolutional layers. These features are processed separately by three specialized expert networks after attention-based weighting. Additionally, an innovative “ heritable loss” function was incorporated to optimize feature similarity, reduce linear correlation, and enhance the model’s multi-task learning capability.

The experimental results demonstrate that the model achieves R² values of 0.94, 0.90, and 0.95 for weight, size uniformity, and quantity predictions, respectively, with NRMSE values of 0.14, 0.33, and 0.19, and NRMSEP values of 0.15, 0.36, and 0.21. Ablation studies reveal that the combination of attention mechanisms, expert networks, and regularization loss significantly enhances prediction accuracy, particularly for weight and quantity estimation. Analysis of the expert networks indicates their different contributions to capturing modality-specific features, with Expert_D_ excelling in weight estimation and Expert_RGB-D_ standing out in size uniformity prediction. Compared to other models, the proposed model excels across all metrics, highlighting its potential for non-destructive quality assessment of strawberries. Future work will focus on optimizing the model architecture and applying it to larger datasets to improve its applicability in real production environments.

## Data Availability

The original contributions presented in the study are included in the article/supplementary material. Further inquiries can be directed to the corresponding author.
